# Comparison of the two intestinal anastomosis methods in pediatric patients

**DOI:** 10.1515/med-2024-1055

**Published:** 2025-08-29

**Authors:** Defeng Zeng, Bingshan Xia, Qianyang Liu, Guoqiang Chen, Kai Gao, Chengwei Yan, Gongli Chen, Hai Zhou, Wen Tang, Chunbao Guo

**Affiliations:** Department of Pediatric Surgery, Women and Children’s Hospital, Chongqing Medical University, Chongqing, 401147, P. R. China; Department of Pediatric General Surgery, Children’s Hospital, Chongqing Medical University, Chongqing, P. R. China; Department of Pediatric General Surgery, Chongqing University Three Gorges Hospital, Chongqing, P. R. China; Department of Pediatric Surgery, Chongqing Health Center for Women and Children, Chongqing, P. R. China; Department of Pediatric Surgery, Women and Children’s Hospital, Chongqing Medical University, #120 Longshan Rd. Yubei, Chongqing, 401147, P. R. China

**Keywords:** continuous single-layer anastomosis, interrupted double-layer anastomosis, anastomotic leakage, postoperative complications, overall hospitalization cost

## Abstract

**Background:**

For pediatric patients, there is still controversy regarding the anastomotic technique used for gastrointestinal construction. The study was to evaluate the continuous single-layer (CSL) intestinal anastomosis method compared with the two-layered interrupted anastomosis.

**Methods:**

We retrospectively reviewed the medical records of the eligible patients following CSL anastomosis (*n* = 252) and interrupted double-layer (IDL) anastomosis (*n* = 196). The influences of CSL or IDL anastomosis on perioperative outcomes, including postoperative complications, anastomotic leakage, hospitalization cost, and postoperative hospital stay, were evaluated.

**Results:**

No significant differences were found between the CSL and IDL groups in terms of anastomotic leakage or postoperative complications. CSL anastomosis was related to favorable clinical outcomes, including anastomotic time (11.6 ± 3.8 vs 24.3 ± 5.9 min, *p* < 0.001) and operative time (111.6 ± 48.6 vs 124.1 ± 54.2 min, *p* = 0.041). There was a decrease in inflammation variable (e.g., C-reactive protein) on postoperative day 5 (10.6 ± 5.8 vs 12.8 ± 6.6 mg/L, *p* = 0.032) in patients with CSL anastomoses compared to the IDL group.

**Conclusions:**

The beneficial effects of CSL anastomosis in pediatric patients were demonstrated with respect to anastomotic time, length of postoperative recovery, and cost incurred.

## 1 Introduction

Anastomosis, the surgical connection of two structures, is a critical procedure in digestive tract surgery. It is a delicate process that, if not executed properly, can lead to a range of complications such as leakage of intestinal contents, formation of an abnormal passage or connection known as an intestinal fistula, and the narrowing of the intestinal lumen known as stricture. These complications not only pose significant risks to the patient’s health but also have a profound impact on postoperative recovery and overall surgical outcomes [1–5].

Traditionally, manual double-layer anastomosis has been the standard approach. This method, while effective, is notably complex and labor-intensive, requiring a high level of skill and experience from the surgeon to ensure the best possible results [6–8]. The intricacies of this technique can lead to longer operating times, which may increase the risk of complications and recovery time for the patient. In contrast, single-layer continuous anastomosis is a more streamlined process that has been shown to reduce the time required for anastomosis construction [9–11]. This technique offers the potential for quicker surgeries, which could be beneficial in reducing patient stress and postoperative recovery times. However, its effectiveness, particularly in the pediatric population, has not yet been fully established and warrants further investigation.

In our region, the double-layer-interrupted anastomosis has been the prevalent method for many years. However, prompted by a comprehensive review of the existing literature, some surgeons at our institute have begun to adopt the continuous single-layer (CSL) anastomosis technique for selected patients [5]. This shift in practice has raised questions about the comparative efficacy of the CSL anastomosis versus the traditional interrupted double-layer (IDL) anastomosis.

To address these questions and provide evidence-based guidance for surgical practice, we undertook a retrospective review of clinical data from patients who underwent intestinal anastomosis at our institute. Our aim was to compare the efficacy of the CSL anastomosis with that of the IDL anastomosis across various parameters, including the incidence of postoperative complications, the duration of surgery, and the overall impact on patient recovery. This analysis is crucial for informing future surgical approaches and improving patient outcomes in pediatric digestive tract surgeries.

## 2 Methods

### 2.1 Patients

The patients who underwent intestinal anastomosis between August 2015 and August 2022 in our institutions were retrospectively reviewed in line with STROBE (Strengthening the Reporting of Observational Studies in Epidemiology) guidelines.

The patients had to meet the following inclusion criteria: age >1 and <16 years; emergent or elective surgeries; and no steroid or immunosuppressive medication administration. We excluded the cases for the stomach and the rectum anastomosis. There were six operative surgeons involved in the current management, each with more than 5-year experiences of intestinal construction.

The baseline data and clinical data of all the patients included were carefully reviewed and recorded. The Institutional Review Board (IRB No. 05-2020-169) of Chongqing Health Center for Women and Children gave expedited approval for the current research under the protection of personal information.

### 2.2 Anastomosis methods

**Figure 1:**
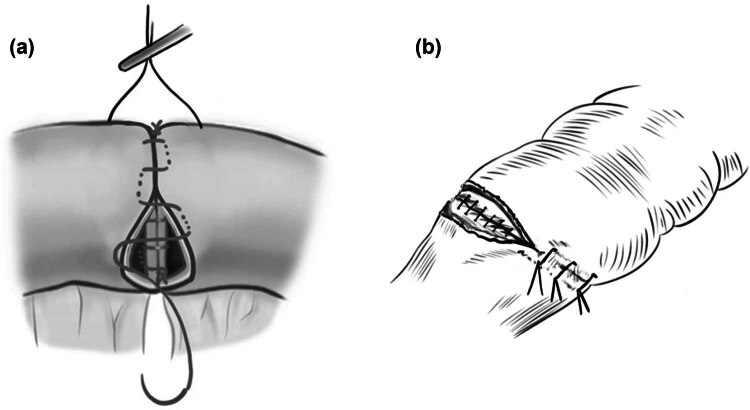
(a) The intestinal wall was constructed using the one-layer continuous horizontal mattress anastomosis from the mesenteric border. The stitches include all layers. (b) The intestinal wall was constructed using an IDL with all the layers incorporated.

In my institute, we have performed the current anastomosis using two methods, in part one, they persist in silk sutures and double-layer interrupted sutures anastomosis for intestinal anastomoses. In another part, we have upgraded this method using polydioxanone (PDS) and single-layer anastomosis. This means that the two methods were used at the same time in similar patient populations by different attending surgeons: the IDL and the CSL for the intestinal construction. The CSL anastomosis began at the mesenteric border with all layers incorporated using a 5–0 PDS suture (Ethicon Inc., Norderstedt, German). The 3–5 mm of needle pitch and edge distance were needed for each bite. For the IDL suture, the inner mucosal layer was sutured using a 5–0 silk thread in an interrupted manner and the outer seromuscular was stitched continuously. To record the anastomosis times, we have recorded ten patients with the respective method. The anastomosis time is defined from the first stitch to cutting the thread. The suturing technique is shown in the Figure 1.

### 2.3 Data collection and outcomes

Comprehensive medical data were collected, encompassing preoperative variables, intraoperative details, and postoperative outcomes. Additional data concerning the timing of surgery (early versus late), the interval between diagnosis and surgery, the presence of preoperative incarceration, and surgical complications (incarceration, surgical injury, postoperative infection, and apnea) were also evaluated. Recurrence rates were likewise examined.

Telephone interviews were conducted from the time of presentation to 8 months post-surgery to monitor perioperative and postoperative complications, such as incarceration, surgical injury, postoperative infection, and apnea. The diagnosis of inguinal hernia and its incarceration was clinically established and verified through surgical exploration. A preterm patient was defined as one born before 37 weeks of gestational age.

The first outcome was the incidence of anastomotic leak. Secondary outcomes included the postoperative outcomes, the postoperative gastrointestinal recovery measures, overall hospitalization cost, and length of postoperative hospital stay. Gastrointestinal function measures included the postoperative nausea or vomiting, the flatus or defecation, and duration for normal diet within the first postoperative 5 days. The Clavien–Dindo classification system was used for the postoperative complications ranking. The hospitalization cost included the cost of an overnight bed, the cost of a pediatric intensive care unit bed, the cost of suture materials, the cost of operating theatre time, and so on.

### 2.4 Statistical analysis

The SPSS version 19.0 (SPSS Inc., Chicago, IL, USA) software was used to perform the statistical analyses. The baseline characteristics, including demographic and preoperative clinical data, were first measured to compare the selection biases. Then, the primary endpoint and secondary endpoints were subjected to statistical comparisons. Continuous data were expressed either as medians (interquartile ranges [IQR]) for unevenly distributed data or the means ± (standard deviations [SD]) for normally distributed data, which were analyzed with the Mann–Whitney *U* test or Student’s *t*-test, respectively. Categorical data were presented as frequencies (percentages) and tested using the chi-square test or Fisher’s exact test as appropriate. The statistical significance was considered if *p* value <0.05.


**Informed consent:** The need for patient consent was waived because this was an observational study with no interventions other than routine care.
**Ethical approval:** The Institutional Review Board approval from Chongqing Children’s Hospital was obtained on August 25, 2017.

## 3 Results

Four hundred and forty-eight patients were eligible for the study, 252 (56.3%) underwent a CSL anastomosis, and 196 (43.7%) underwent an IDL anastomosis. The two groups were matched with regard to the baseline and surgical features, including the location of the anastomosis (Table 1).

**Table 1:** Baseline demographics of eligible patient and preoperative variables

Suture	CSL (252)	IDL (196)	*p* values
Age (years), median (IQR)	3.8 (1.2–6.8)	3.7 (1.1–7.1)	0.34
Male:female	104:148	86:110	0.32
**Comorbidities,** ***N*** **(%)**			
Shock	28	20	0.44
Preoperative anemia (Hb < 12 g/dL)	89	65	0.35
Length of intestinal resection (cm), median (IQR)	12.2 (3.5–31.8)	11.8 (3.8–29.7)	0.28
**Location of the anastomosis,** ***N*** **(%)**			
Small bowel resection and anastomosis	193 (76.6)	151 (77.0)	0.50
Ileum to colon anastomosis	59 (23.4)	45 (23.0)	
**Operative indications,** ***N*** **(%)**			
Intussusception	98 (38.9)	75 (38.3)	0.49
Adhesive small bowel obstruction	49 (19.4)	41 (20.9)	0.39
Meckel’s diverticulum	67 (26.6)	49 (25.0)	0.39
Incarcerated inguinal hernia	38 (15.1)	31 (15.8)	0.47

Abbreviations: CSL = continuous single layer; IDL = interrupted double layer.

The postoperative outcomes between the two groups are illustrated in Table 2. The total operative time and the mean time for anastomosis construction in the CSL group were significantly shorter than that of the IDL group.

**Table 2:** Surgical outcomes in the matched population

Suture	CSL (252)	IDL (196)	*p* values
EBL (mL), mean ± SD	11.4 ± 6.8	12.5 ± 7.4	0.41
Transfused patients, *N* (%)	53 (21.0)	39 (19.9)	0.38
Durations of parenteral nutrition, days, mean ± SD	2.4 ± 1.5	3.1 ± 1.2	0.061
Albumin (g/L, normal range, 35–50)	32.9 ± 7.6	31.8 ± 6.9	0.19
CRP at POD 5 (mg/L, normal value: 0–8)	10.6 ± 5.8	12.8 ± 6.6	0.032
Overall hospitalization cost (USD), mean ± SD	3791.5 ± 1329.9	3886.7 ± 1413.8	0.09
Postoperative hospital stay, days	8.6 ± 1.4	9.1 ± 1.7	0.09
Operative time (min), mean ± SD	111.6 ± 48.6	124.1 ± 54.2	0.041
Anastomosis time (min), *n* = 10	11.6 ± 3.8	24.3 ± 5.9	<0.001
Anastomosis cost (USD), *n* = 10	35.8	3.1	<0.001

Abbreviations: CRP = C-reaction protein; CSL = continuous single layer; EBL = estimated blood loss; IDL = interrupted double layer; POD = postoperative day.

The overall hospitalization cost in the CSL group was approximately 3791.5 ± 1329.9 USD, whereas in the IDL group, it was 3886.7 ± 1413.8 USD (*p* = 0.09, Table 2); however, for the anastomosis cost, it was higher for CSL anastomoses (35.8 USD) (1 pack of 5–0 PDS suture) than for IDL anastomoses (3.1 USD) (*p* < 0.001). Significant differences were also found in terms of postoperative inflammation variables (e.g., C-reactive protein at postoperative day 5) between the two groups.

No statistically significant differences were indicated in the total episodes of complications, postoperative anastomotic leakage, wound infection, sepsis, and peritonitis or abscess between the two groups, all of which might be associated with the surgical intervention (Table 3). Only six patients in the CSL group reported reoperation versus seven patients in the IDL group (*p* = 0.32) (Table 3). We further conducted a stratified analysis of the results of small intestine anastomosis alone, and the results are listed in Table 4, which are consistent with the current conclusions.

**Table 3:** Postoperative complications in the matched population (chi-square test)

Suture	CSL (252)	IDL (196)	*p* value
Total complications (at least 1 complication), *N* (%)	49 (19.4)	42 (21.4)	0.34
Surgical wound infection, *N* (%)	18 (7.1)	16 (8.2)	0.41
Pneumonia, *N* (%)	12 (4.8)	11 (5.6)	0.42
Sepsis, *N* (%)	3 (1.2)	4 (2.0)	0.37
Peritonitis or abscess, *N* (%)	15 (6.0)	13 (6.6)	0.46
Anastomotic leakage, *N* (%)	2 (0.8)	3 (1.2)	0.38
Anastomotic stricture	0	1	0.44
Hospital readmission, *N* (%)	21 (8.3)	23 (11.7)	0.15
Re-operation, *N* (%)	6 (2.4)	7 (3.6)	0.32

Abbreviations: CSL = continuous single layer; IDL = interrupted double layer.

**Table 4:** Postoperative complications following small intestine anastomosis (chi-square test)

Suture	CSL (193)	IDL (151)	*p* value
Total complications (at least 1 complication), *N* (%)	26 (13.5)	23 (15.2)	0.44
Surgical wound infection, *N* (%)	8 (4.1)	6 (4.0)	0.45
Pneumonia, *N* (%)	7 (3.6)	6 (4.0)	0.46
Sepsis, *N* (%)	0 (0)	1 (0.7)	0.38
Peritonitis or abscess, *N* (%)	11 (5.7)	9 (6.0)	0.42
Anastomotic leakage, *N* (%)	0 (0)	0 (0)	0.48
Hospital readmission, *N* (%)	11 (5.7)	12 (7.9)	0.35
Re-operation, *N* (%)	2 (1.0)	3 (2.0)	0.39

Abbreviations: CSL = continuous single layer; IDL = interrupted double layer.

## 4 Discussion

Here, we evaluated the safety and efficacy of the CSL anastomosis method, including the construct time, and anastomotic leak incidence compared with the IDL anastomoses. The CSL anastomosis was used in 252 patients and was more efficient in terms of both anastomotic and overall operative time compared to the IDL technique. The anastomotic leak of CSL anastomosis was lower than that of IDL anastomotic techniques [11,12]. Moreover, the overall hospitalization cost of patients with CSL anastomosis was somewhat reduced.

The ideal target for anastomosis is healing without leakage, which is decided by the skill of the surgeon [13]. Poor techniques are clearly associated with anastomotic failure, with lower leakage incidences for experienced surgeons [14–16]. For blood vessel sutures, the continuous suture is undoubtedly appropriate with the tightest connection. For the mechanical integrity of small intestine and colon anastomosis, CSL anastomosis was as strong as interrupted suturing [17,18]. In the present pediatric population, the incidence of anastomotic leak following the CSL suture was comparable to those seen in the control group and to the findings in the available literature [18]. The advantage of the continuous suture was that it might contribute to avoid the anastomosis ischemia through the anastomotic diameter adjustment according to the intraluminal forces while rendering the anastomosis water tight [19]. We only record one case of anastomotic strictures for the two anastomotic construction methods, which should be related to the extremely low incidence. Due to excessive tissue inversion of the IDL suture, there might be more risk for the lumen narrowing and stricture formation [20], the weakness should be avoided with one layer of sutures in the CSL technique.

Additionally, for continuous anastomosis, the edges of the intestine were pruned with an adequate blood supply [21,22]. Previous research indicated that from the single-layer to the two-layer manual stapled suture, the blood flow for the suture line was decreased. Although more hemostatic effect was associated with the double-layer technique, mucosa strangulation should also be touched off with low blood flow, which should be avoided through CSL technique as sutures are stitched while sparing the mucosa [23]. The recovery from the inflammatory infiltrate status might be reflected by the postoperative C-reactive protein (CRP). Here, we indicated that there was a slight decrease in the CSL group, although we did not know that the beneficial effects should be attributed to the CSL anastomosis. In the experimental research, the CSL anastomosis method was favored for the reduction of inflammation and fibroblast cells compared with the IDL method, which might be associated with the recovery of gastrointestinal function [24].

The operation and anesthesia time were the most priority points for all the surgeries. For the current research, due to the majority of procedures being emergency operations, it should be beneficial to quicken the gastrointestinal construction to reduce the whole surgical time [25]. The CSL anastomosis technique is so easy with a greatly shortened length of anastomosis time, which should be associated with the overall operation time reduction. A similar finding for comparison with the length of anastomosis time was indicated in another report [26]. Although the materials cost for the CSL anastomoses was higher than that for the IDL anastomosis, the total operation cost was the same or lower for the CSL anastomoses. We think that this might be attributed to the rapid recovery from the CSL anastomoses, following the shorter operation time and the shorter hospital stay.

The current research has several weaknesses that need to be considered. First, the findings of this study did not result from the random assignment of participants to each group. Moreover, a long time period in which practice for many patients may not reflect the current treatment algorithms, leading to an inherent risk of selection bias among study patients. The CSL anastomoses were more likely to initiate in recent years for the patients who needed intestinal construction. Therefore, the current findings should be interpreted with caution.

## 5 Conclusions

In summary, the present research compared the safety and efficacy of CSL anastomosis with IDL anastomosis in pediatric patients, suggesting that compared to its conventional counterpart, the CSL anastomosis technique is more efficient in terms of anastomosis time and overall surgical time and has a lower incidence of anastomotic leakage compared to the IDL technique. Considering the duration of the anastomosis procedure and its easy learning curve, CSL may be reliably introduced and prove the optimal choice in most pediatric surgical settings. A prospective randomized trial should be taken to verify these findings.

## Abbreviations

CRP: C-reactive protein
CSL: continuous single layer
EBL: estimated blood loss
IDL: interrupted double layer
POD: postoperative days

## References

[1] Slieker JC, Daams F, Mulder IM, Jeekel J, Lange JF. Systematic review of the technique of colorectal anastomosis. JAMA Surg. 2013;148:190–201. 10.1001/2013.jamasurg.33 23426599

[2] Law WL, Bailey HR, Max E, Butts DR, Smith KW, Thompson DA, et al. Single-layer continuous colon and rectal anastomosis using monofilament absorbable suture (Maxon): Study of 500 cases. Dis Colon Rectum. 1999;42:736–40. 10.1007/BF02236928 10378597

[3] Cikot M, Kones O, Gedikbası A, Kocatas A, Karabulut M, Temizgonul KB, et al. The marker C-reactive protein is helpful in monitoring the integrity of anastomosis: plasma calprotectin. Am J Surg. 2016;212:53–61. 10.1016/j.amjsurg.2015.06.018 26606896

[4] Masud D, Undre S, Darzi A. Using manual dexterity to predict the quality of the final product in the small bowel anastomosis after a period of training. Am J Surg. 2012;203:776–81. 10.1016/j.amjsurg.2011.06.054 22221995

[5] Louridas M, Szasz P, de Montbrun S, Harris KA, Grantcharov TP. Can we predict technical aptitude?: A systematic review. Ann Surg. 2016;263:673–91. 10.1097/SLA.0000000000001283 26079898

[6] Datta V, Bann S, Mandalia M, Darzi A. The surgical efficiency score: A feasible, reliable, and valid method of skills assessment. Am J Surg. 2006;192:372–8. 10.1016/j.amjsurg.2006.06.001 16920433

[7] Moriura S, Kobayashi I, Ishiguro S, Tabata T, Yoshioka Y, Matsumoto T. Continuous mattress suture for all hand-sewn anastomoses of the gastrointestinal tract. Am J Surg. 2002;184:446–8. 10.1016/s0002-9610(02)00999-6 12433611

[8] Maan ZN, Maan IN, Darzi AW, Aggarwal R. Systematic review of predictors of surgical performance. Br J Surg. 2012;99:1610–21. 10.1002/bjs.8893 23034658

[9] Wilasrusmee C, Phromsopha N, Lertsitichai P, Kittur DS. A new vascular anastomosis model: Relation between outcome and experience. Eur J Vasc Endovasc Surg. 2007;33:208–13. 10.1016/j.ejvs.2006.09.026 17097903

[10] Cossu ML, Coppola M, Fais E, Ruggiu M, Spartà C, Profili S, et al. The use of the Valtrac ring in the upper and lower gastrointestinal tract, for single, double, and triple anastomoses: A report of 50 cases. Am Surg. 2000;66:759–62. 10966036

[11] Burch JM, Franciose RJ, Moore EE, Biffl WL, Offner PJ. Single-layer continuous versus two-layer interrupted intestinal anastomosis: a prospective randomized trial. Ann Surg. 2000;231:832–7. 10.1097/00000658-200006000-00007 PMC1421072 10816626

[12] Ordorica-Flores RM, Bracho-Blanchet E, Nieto-Zermeño J, Reyes-Retana R, Tovilla-Mercado JM, Leon-Villanueva V, et al.Intestinal anastomosis in children: A comparative study between two different techniques. J Pediatr Surg. 1998;33:1757–9. 10.1016/s0022-3468(98)90279-2 9869045

[13] Oláh A, Belágyi T, Neuberger G, Gamal EM. Use of different absorbable sutures for continuous single-layer anastomosis in the gastrointestinal tract. A prospective, randomized study. Dig Surg. 2000;17:483–5. 10.1159/000051944 11124552

[14] Kim SH, Choi HJ, Park KJ, Kim JM, Kim KH, Kim MC, et al. Sutureless intestinal anastomosis with the biofragmentable anastomosis ring: experience of 632 anastomoses in a single institute. Dis Colon Rectum. 2005;48:2127–32. 10.1007/s10350-005-0144-3 16228843

[15] Hussain A, Mahmood H, Nicholls J, El-Hasani S. Continuous figure-of-eight suturing in upper and lower gastrointestinal anastomosis. Singap Med J. 2008;49:672–5. 18830539

[16] Max E, Sweeney WB, Bailey HR, Oommen SC, Butts DR, Smith KW, et al. Results of 1,000 single-layer continuous polypropylene intestinal anastomoses. Am J Surg. 1991;162:461–7. 10.1016/0002-9610(91)90262-c 1951910

[17] Sandrasegaran K, Maglinte DD, Lappas JC, Howard TJ. Small-bowel complications of major gastrointestinal tract surgery. AJR Am J Roentgenol. 2005;185:671–81. 10.2214/ajr.185.3.01850671 16120916

[18] Herrle F, Diener MK, Freudenberg S, Willeke F, Kienle P, Boenninghoff R, et al. Single-layer continuous versus double-layer continuous suture in colonic anastomoses-a randomized multicentre trial (ANATECH Trial). J Gastrointest Surg. 2016;20:421–30. 10.1007/s11605-015-3003-0 26525206

[19] Bailey HR, LaVoo JW, Max E, Smith KW, Butts DR, Hampton JM. Single-layer polypropylene colorectal anastomosis. Experience with 100 cases. Dis Colon Rectum. 1984;27:19–23. 10.1007/BF02554066 6360594

[20] Park KJ, Woo JS, Jeong SS, Yi JH. Continuous “over and over” suture for tricuspid ring annuloplasty. Korean J Thorac Cardiovasc Surg. 2012;45:19–23. 10.5090/kjtcs.2012.45.1.19 PMC3283779 22363903

[21] Close K, Epstein KL, Sherlock CE. A retrospective study comparing the outcome of horses undergoing small intestinal resection and anastomosis with a single layer (Lembert) or double layer (simple continuous and Cushing) technique. Vet Surg. 2014;43:471–8. 10.1111/j.1532-950X.2014.12143.x 24689880

[22] Orsay CP, Bass EM, Firfer B, Ramakrishnan V, Abcarian H. Blood flow in colon anastomotic stricture formation. Dis Colon Rectum. 1995;38:202–6. 10.1007/BF02052452 7851178

[23] Garude K, Tandel C, Rao S, Shah NJ. Single layered intestinal anastomosis: A safe and economic technique. Indian J Surg. 2013;75:290–3. 10.1007/s12262-012-0487-7 PMC3726811 24426455

[24] Li GC, Xu Y, Zhang YC, Zhang FC, Wang Q, Ma QJ. Efficacy of single-layer continuous suture of the posterior wall in anastomosis involving a difficult location of the digestive tract. Oncol Lett. 2014;8:1567–74. 10.3892/ol.2014.2397 PMC4156267 25202369

[25] Shogan BD, Carlisle EM, Alverdy JC, Umanskiy K. Do we really know why colorectal anastomoses leak? J Gastrointest Surg. 2013;17:1698–707. 10.1007/s11605-013-2227-0 23690209

[26] Khan RAA, Hameed F, Ahmed B, Dilawaiz M, Akram M. Intestinal anastomosis: Comparative evaluation for safety, cost effectiveness, morbidity and complication of single versus double layer. Professional Med J. 2010;17:232–4.

